# Prognostic factors on survival rate of fingers replantation

**DOI:** 10.1590/1413-78522015230101026

**Published:** 2015

**Authors:** José Queiroz Lima, Alberto De Carli, Hugo Alberto Nakamoto, Gustavo Bersani, Bruno Eiras Crepaldi, Marcelo Rosa de Rezende

**Affiliations:** Universidade de São Paulo, Faculdade de Medicina, Hospital das Clínicas, São Paulo, SP, Brazil, Instituto de Ortopedia e Traumatologia do Hospital das Clínicas da Faculdade de Medicina da Universidade de São Paulo, São Paulo, SP, Brazil

**Keywords:** Replantation, Finger injuries, Amputation, traumatic

## Abstract

**Objective:**

To evaluate the factors that influence the survival rate of replantation and revascularization of the thumb and/or fingers.

**Methods:**

We included fifty cases treated in our department from May 2012 to October 2013 with total or partial finger amputations, which had blood perfusion deficit and underwent vascular anastomosis. The parameters evaluated were: age, gender, comorbidities, trauma, time and type of ischemia, mechanism, the injured area, number of anastomosed vessels and use of vein grafts. The results were statistically analyzed and type I error value was set at p <0.05 .

**Results:**

Fifty four percent of the 50 performed replantation survived. Of 15 revascularizations performed, the survival rate was 93.3%. The only factor that affected the survival of the amputated limb was the necessity of venous anastomosis.

**Conclusion:**

We could not establish contraindications or absolute indications for the replantation and revascularization of finger amputations in this study. Level of Evidence III, Retropective Study.

## INTRODUCTION

Amputation is defined as total or partial surgical or traumatic separation of a part of the whole body. The amputation of a finger is a common injury with important consequences and can cause psychological changes, permanent functional deficit and inability to work. Moreover, it also brings large direct and indirect financial loss to the patient and to the society.^1^^-^ 3 Most of these injuries occur in the workplace, especially for the male population at productive ages.^4^^-^ 6 In a study based on the NTDB (National Trauma Databank) from 2000 to 2004 6,155 patients underwent finger amputation in the US, accounting for 69.1% of amputations.^5^ There is no national data in Brazil on the incidence of traumatic amputations of fingers.

Amputations can be divided into complete or incomplete. In complete amputations, the portion of the injured member is completely separated from the proximal stump. Incomplete amputations are those in which, although presenting a connection to the amputated portion, there is a need for anastomosis, at least of one artery to maintain the viability of the member.^7^

With the advent of microsurgical techniques,^8^ reimplantation emerged as an alternative for the treatment of these lesions. Reimplantation of a member experimentally in animal model was successfully held at the beginning of the 20th Century.^8^^-^ 11 However, the first replantation of a human upper limb was performed only in the 60s. In 1962, Malt and McKhann^12^ reimplanted the arm of a 12 year old child. Tamai and Komatsu,^13^ in 1968, firstly reported a microsurgical replantation of a finger. Since then, several centers around the world organized themselves in order to provide proper treatment to amputees, with success rates exceeding 50%.^14^^-^ 17

## MATERIALS AND METHODS

This is a retrospective study to evaluate the factors influencing the survival of replantation and revascularization of thumb and/or other fingers. This article was approved by the Institution's Ethics Committee in accordance with the protocol No. 759,220.

This study comprised all patients treated from May 2012 to October 2013 at our department with partial or total amputation of thumbs or fingers, involving Verdan areas^18^ 1, 2 or 3 that had blood perfusion deficit and vascular anastomoses to maintain the viability of the injured part. The study excluded those who, despite having been indicated for reimplantation, it did not take place due to lack of local conditions or failure to present distal perfusion after arterial anastomosis.

Data were collected from medical records of patients undergoing replantation, revascularization and/or regularization of amputations. In the results, we assessed the correlation between finger survival of those who were submitted to reimplantation and/or revascularization and the patients' factors related to trauma and surgical procedure. (Table 1)

**Table 1.** Factors evaluated to determine reimplantation survival and revascularization of thumb and/or fingers.

Patient related factors	Trauma related factors	Surgical procedure related factors
Age	Mechanism	Number of anastomosed arteries
Comorbidities	Cutting injuries	Number of anastomosed veins
Systemic Hypertension	Crushing	Use of venous graft
Habits (Smoking)	Avulsion	
	Ischemia time	
	Ischemia type: hot or cold	
	Injury zone (Verdan) Osteoarticular injury location	

For data storage, an Excel^(r)^ spreadsheet for MAC was used. Subsequently, data were imported into SPSS 20.0 for MAC software for statistical analysis. Descriptive statistics was performed and measurements such as mean and standard deviation were used as a degree of central tendency and variability of the data. The Kolmogorov-Smirnov test was used to test the distribution of the data. For analysis of inferential statistics, logistic regression models were used with the stepwise method, so in every step of the procedure the most important variable, in statistical terms, was the one that produced the biggest change in log-likelihood in relation of the model that did not contain the variable. The value accepted as the type I error was p≤0.05.

## RESULTS

**Figure 1.**
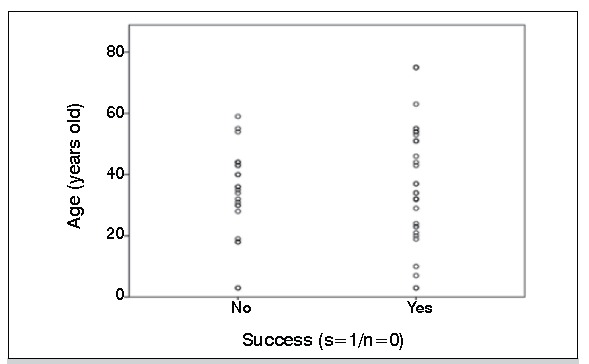
Mean age of patients.

During the study period, 50 reimplantation and 15 revascularizations were performed in 45 patients. Thirty-five patients underwent reimplantation/revascularization of one finger, three of two fingers, five of three fingers, one of four fingers and one of five fingers. The mean age of patients was 36 years old (range 3-75). (Figure 1) Of these patients, two (4.4%) were women and 43 (95.6%) were men. Cutting injuries were the main mechanism of injury in 54 (83%) cases, three (4.6%) were avulsion injuries and eight (12.3%) were crush injuries. The majority (73.3%) of patients had lesions in the non-dominant hand. Of the 65 procedures performed, 28 (43%) involved the thumb, 11 (16.9%) the index finger, 12 (18.4%) the middle finger, nine (13.8%) the ring finger and five (7.6 %) the little finger.

**Figure 2.**
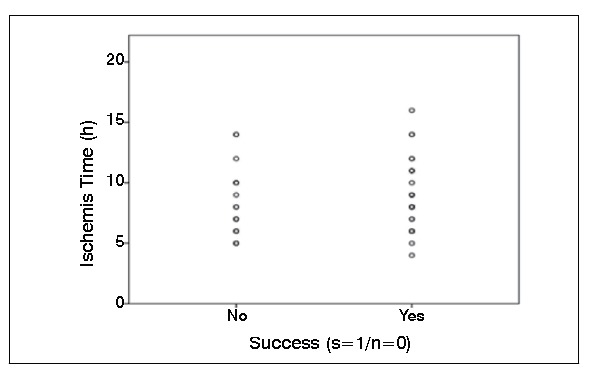
Mean ischemia times.

Workplace accidents corresponded to 55.6% of patients and the majority worked in civil construction (48.8%) or the industry (13.3%). The average ischemia time was 8.39 hours, 7.57 hours in the fingers that did not survive and nine hours in successful reimplantation/revascularization. (Figure 2) Nine (20%) patients were smokers and five (11.1%) patients had hypertension. No replantation or revascularization was performed in patients with a history of type II diabetes mellitus, alcoholism or psychiatric illness.

Regarding the injured area of the fingers, 44 (67.7%) of the fingers were injured in Verdan zone 2, 14 (21.5%) in zone 1 and seven (10.8%) in zone 3. Twenty-eight presented simple fractures trait, 31 were comminuted and six pure dislocations without bone injury. Eight amputations had metacarpal fractures, 29 in the proximal phalanx, 10 on medial phalanx and one on the distal phalanx. Ten had fracture-dislocation at the proximal inter-phalangeal level and six at the distal inter-phalangeal.

Among the 50 reimplantation performed, only 20 had a reconstructed vein and on 30 cases there were two or more venorrhaphies. In all cases, only one arterial anastomosis was performed. Sixteen replantation required vascular graft for arterial anastomosis and no finger submitted to revascularization required venous graft.

Of the data analyzed, the only one that showed a statistically significant difference was the need for venous anastomosis. Of the 50 reimplantations performed 27 (54%) fingers survived. Of the 15 revascularization performed, only one did not survive, which represents a 93.3% survival rate. (Table 2)

**Table 2.** Need to perform venous anastomosis and reimplantation success.

Need of venorrhaphy	Reconstructed fingers	Success (n)	Success (%)	Statistical Test	P value	
Revascularizations	15	14	93.3%	Fisher's Exact Test	0.006	0.004
Reimplantation	50	27	54%			

## DISCUSSION

Given an amputation case, many factors are determinant to make the decision whether to reimplant it or not. We must consider what are the real chances of success of the procedure in terms of survival and functional recovery for actually indicate reimplantation. Therefore, there is a need to define objective parameters that can signal to the prognostic factors of this procedure. (Table 3)

**Table 3.** Surgical technique employed and success of procedure.

Surgical Technique	Number of reconstructions	Number of successful reconstructions	Successful reconstructions (%)	Statistical test	P value
Number of reconstructed veins					
One	20	8	40%	Pearson chi-square = 2.630	0.105
Two or more	30	19	63.3%		
Graft for arteriorrhaphy					
Yes	16	9	56.25%	Fisher's Exact Test	0.560
No	49	32	65.3%		
Need of venorrhaphy					
Revascularizations	15	14	93.3%	Fisher's Exact Test	0.006
Reimplantation	50	27	54%		

Current literature suggests some indications for reimplantation, such as thumb amputations, multiple fingers amputations, partial hand amputation, any level of amputations in children, wrist or forearm amputation or single distal finger insertion of the superficial flexor of the finger.^19^^-^ 25

The following conditions are considered relative contraindications for reimplantation, because of their lower survival rates and/or poor functional outcome: crush injuries or member avulsion, amputations at multiple levels, prolonged ischemia, single finger amputation proximal to the finger superficial flexor insertion (mainly index and little finger), amputations in patients with systemic diseases or associated severe injury, severe atherosclerosis and psychiatric patients without proper treatment.^19^^,^ 24 ^-^ 27

The anastomosis of two or more veins showed a higher survival rate than when only one vein was sutured, however, the result was not statistically significant (p=0.105). (Table 3)

Although several studies^24^^-^ 27 have shown higher failure rates in anastomoses of replantation in smokers, this association was not observed in our study group (p=0.267). (Table 4) Therefore, despite data showing the negative effects of smoking on blood flow, limb replantation can be attempted associated to encourage smoking cessation in the postoperative period.

**Table 4.** Factors evaluated and reimplantation success rate.

Factors	Reconstructed fingers	Success (n)	Success (%)	Statistical Test	P value
Smoking					
Yes	15	11	73.3%	Fisher's Exact Test	0.543
No	50	30	60%		
Hypertension					
Yes	8	7	87.5%	Fisher's Exact Test	0.240
No	57	34	59.6%		
Amputated finger					
Thumb	28	14	50%	Pearson chi-square=7.317	0.120
Index	11	7	63.6%		
Medium finger	12	10	83.3%		
Ring finger	9	5	55.5%		
Minimum finger	5	5	100%		
Verdan zone					
I	14	7	50%	Pearson chi-square=1.467	0.480
II	43	29	67.4%		
III	7	4	57.1%		
Osteoarticular injury					
Simple fracture	28	15	53.6%	Pearson chi-square=2.433	0.296
Comminuted fracture	31	21	67.7%		
Pure sprain	6	5	83.3%		
Trauma mechanism					
Cutting	54	33	61.1%	Pearson chi-square=0.595	0.743
Crushing	8	6	75%		
Avulsion	3	2	66.6%		
Fracture/sprain location					
Metacarpal	8	5	62.5%	Pearson chi-square=4.133	0.530
Proximal phalanx	29	16	55.2%		
Medial phalanx	10	7	70%		
Distal phalanx	1	0	0%		
Inter-proximal phalangeal	10	8	80%		
Inter-distal halangeal	7	5	71.4%		

## CONCLUSION

In this study, possibly due to the low number of cases, the only factor that directly affected the survival of the amputated limb was the need of venous anastomosis. Therefore, it was not possible to establish contraindications or absolute indications for reimplantation/revascularization aiming at survival of the amputated finger.
